# Achieving food security for one million sub-Saharan African poor through push–pull innovation by 2020

**DOI:** 10.1098/rstb.2012.0284

**Published:** 2014-04-05

**Authors:** Zeyaur R. Khan, Charles A. O. Midega, Jimmy O. Pittchar, Alice W. Murage, Michael A. Birkett, Toby J. A. Bruce, John A. Pickett

**Affiliations:** 1 Habitat Management Programme, International Centre of Insect Physiology and Ecology (icipe), PO Box 30, Mbita Point, Kenya; 2 Department of Biological Chemistry and Crop Protection, Rothamsted Research, Harpenden AL5 2JQ, UK

**Keywords:** food security, pests, climate change, push–pull technology, sub-Saharan Africa

## Abstract

Food insecurity is a chronic problem in Africa and is likely to worsen with climate change and population growth. It is largely due to poor yields of the cereal crops caused by factors including stemborer pests, striga weeds and degraded soils. A platform technology, ‘push–pull’, based on locally available companion plants, effectively addresses these constraints resulting in substantial grain yield increases. It involves intercropping cereal crops with a forage legume, desmodium, and planting Napier grass as a border crop. Desmodium repels stemborer moths (push), and attracts their natural enemies, while Napier grass attracts them (pull). Desmodium is very effective in suppressing striga weed while improving soil fertility through nitrogen fixation and improved organic matter content. Both companion plants provide high-value animal fodder, facilitating milk production and diversifying farmers’ income sources. To extend these benefits to drier areas and ensure long-term sustainability of the technology in view of climate change, drought-tolerant trap and intercrop plants are being identified. Studies show that the locally commercial brachiaria cv mulato (trap crop) and greenleaf desmodium (intercrop) can tolerate long droughts. New on-farm field trials show that using these two companion crops in adapted push–pull technology provides effective control of stemborers and striga weeds, resulting in significant grain yield increases. Effective multi-level partnerships have been established with national agricultural research and extension systems, non-governmental organizations and other stakeholders to enhance dissemination of the technology with a goal of reaching one million farm households in the region by 2020. These will be supported by an efficient desmodium seed production and distribution system in eastern Africa, relevant policies and stakeholder training and capacity development.

## 1. Introduction

Africa faces serious challenges in feeding its population, having reverted from being a net exporter of agricultural commodities to being a net importer of the same for the last three decades. Indeed, Food and Agriculture Organization (FAO) statistics reveal that the amount imported is increasing at an almost exponential level [1]. The continent also has the highest population growth rates in the world. Human population more than tripled in the second half of the twentieth century, from 230 million to 811 million [2]. In spite of this rapid surge in human population, average growth in food production in the continent has at best stagnated, with reports indicating decline in crop yields over the last few decades in several places within the continent [3]. Indeed, Africa has the tragic distinction of being the only continent where food production has been declining in the past few decades.

While there have been increases in *per capita* food production elsewhere (e.g. East Asia and Pacific, and Latin America by 30% and 20%, respectively), there has been an annual decline of at least 3% in *per capita* food production in sub-Saharan Africa (SSA) since 1990 [3]. Indeed, about 33% of people in the region are undernourished, with more than 60% of the undernourished being in eastern Africa [3].

One of the main causes of the chronic food insecurity witnessed in Africa is poor crop yields, largely caused by insect pests, weeds and degraded soils. This is complicated further by the increasingly hot and dry weather conditions associated with climate change [4,5]. Over 75% of arable land in Africa is degraded, a result of continuous cropping with minimal or no investment in soil improvement or even maintenance. Increasing crop production is thus an important challenge in addressing economic growth, alleviating poverty and arresting environmental degradation over most of SSA [6]. Cereals, including maize (*Zea mays* L.) and sorghum (*Sorghum bicolor* (L.) Moench), are the most important food and cash crops for millions of rural farm families in the predominantly mixed crop-livestock farming systems of SSA [6]. The efficient production of cereals, per unit of input, is therefore central to the food security challenge.

## 2. Biotic constraints to cereal production–pest problems

Smallholder cereal production is severely constrained by insect pests and the parasitic weeds in the genus *Striga* (Orobanchaceae), commonly referred to as striga. Among the 21 economically important lepidopteran stemborers in Africa [7], the indigenous *Busseola fusca* Füller (Noctuidae) and the invasive *Chilo partellus* Swinhoe (Crambidae) are the most devastating in SSA [8]. Damage is caused by the larval stages of the stemborers whose feeding results in yield losses of up to 88%, depending on the crop cultivar, developmental stage of the plant at infestation, infestation rate and prevailing environmental conditions, among other factors [8]. Although certain insecticides are recommended for control of these pests by the National Agricultural Research Systems in SSA, complete control is seldom achieved, and, more importantly, the resource-poor farmers cannot afford such chemical treatments.

There are about 23 species of striga in Africa, out of which *Striga hermonthica* (Del.) Benth. and *Striga asiatica* (L.) O. Kuntz, are the most important [9,10]. Striga are obligate root parasites of cereal crops that inhibit normal host growth via three processes: competition for nutrients, impairment of photosynthesis [11] and a phytotoxic effect within days of attachment to the hosts [12,13]. Such stresses can modify the nutritional value of the plant to herbivores, its ability to tolerate insect attack and the insects’ responses on the plant [14]. Indeed, maize plants infested by striga were found to be preferred for oviposition by stemborer moths relative to uninfested plants [15]. Infestation by striga causes up to 100% yield loss and over SSA annual losses estimated at $40.8 million [16]. These effects are more serious under conditions of poor soil fertility [17], with nitrogen and phosphorus deficiency being the most serious in accentuating the severity of damage to the host plants [18]. Unfortunately, the problem of striga is continuing to extend to new areas in SSA as farmers abandon heavily infested fields for new ones [19,9].

In spite of the serious crop losses associated with striga infestation, effective control of the weed has been elusive. Reasons for this range from the fact that striga is highly prolific, with an individual plant producing thousands of tiny dust-like seeds that can remain viable in the soil for over 10 years [20,21]. It also has a complicated mode of parasitism, with vascular connections to the host occurring below ground. Moreover, most of the damage to the host plant is caused by the subterranean development stage of the parasite following its germination [22], a process that is induced by signalling molecules including strigolactones that form part of the exudates of host and some non-host plants [23–25]. For effective control of striga, key principles should include reducing the seed bank in the soil, preventing new seed production and spread from infested to non-infested soils, and improving soil fertility. Efforts to control the weed thus far have only reported limited and localized success and with limited uptake owing to biological and socio-economic reasons [17]. A recent technology for controlling striga through imapazyr herbicide-tolerant mutant maize (IR maize) has shown significant increase in maize yields [26]. However, success of IR maize will depend on how widely it is adopted by resource-poor farmers in striga-infested areas because it involves buying hybrid seeds every cropping season, a practice not generally employed by smallholder farmers in SSA because they save their own seed. There are also the challenges of herbicide seed treatment, for example the cost and handling of the treated seeds, and occasional poor emergence of maize with both limited and very high rainfall at germination [27].

## 3. Abiotic and associated socio-economic constraints

Land, the natural resource base for millions of smallholder farmers in SSA, is overexploited because of high incidences and severity of production constraints, purchased inputs are scarcely used or absent and environmental factors too erratic for secure investment in inputs. Conservative estimates indicate that about two-thirds of agricultural land in the region is degraded [28], with most of this degradation owing to nutrient depletion, inadequate organic matter and wind erosion. These are exacerbated by overgrazing and improper agricultural practices. This degradation has been increased further by invasion of farmland by persistent weeds, for example striga, resulting in declining crop yields. With increasing human population and the need to increase food production, in the face of declining land sizes available for production, further expansion and intensification of food production could also have a potentially degrading effect on the environment. In SSA, the most conspicuous symptoms of the negative impact of land degradation on food production are stagnating and declining yields and increasing levels of poverty. The threat of degradation may also be reflected by the need to use a higher level of inputs in order to maintain yields, which is not an option for the majority of the resource-poor smallholder farmers in the region. There is therefore a need for measures to check land degradation and bring the degraded lands back to productivity through adoption of appropriate farming systems.

## 4. Climate change

The food security situation in most of SSA is further threatened by climate change which is expected to have far reaching effects on cereal production. Indeed, projections indicate that unless drastic steps are taken, SSA will have more than 500 million food insecure people by 2020 [29]. Climate change is anticipated to have far reaching effects on cereal production in SSA, consequently posing a threat to its ability to attain the millennium development goals (MDGs). The magnitude and speed of climate change over major crop areas in the region has been predicted by calculating the percentage overlap between historical (1960–2002) crop growing season temperature range and the projected 2025, 2050 and 2075 values over reported crop area [30]. Results indicate that growing season temperature at any given maize growing region in Africa will overlap on average 58% with its historical observations by 2025, 14% by 2050 and 3% by 2075. This suggests that within two decades, growing season average temperature will be hotter than any year in historical experience for 4 years out of 10 for the majority of African maize areas, growing to nearly 9 out of 10 by 2050 and nearly 10 out of 10 in 2075 [30]. Similar results have been reported for the other cereal crops, with rainfall progressively becoming more unpredictable. These predictions accompany indications that atmospheric temperature and incidences of flood and drought will continue to increase. These will result in progressively more serious land degradation and increased pest and weed pressure, increased incidences of crop failure and general increases in food and nutritional insecurity for resource-poor farmers in many parts of SSA. To adapt to these adverse conditions, there is a need for sustainable intensification of the farming systems with improvements in yields per unit of land together with inbuilt components that improve the ecological integrity of the production systems while mitigating the problems occasioned by the changing climate [31]. Specifically, the resource constrained smallholder farmers will need to move to more drought resistant crops, to small ruminants for dairy production and to employ technologies that improve soil quality. There will also be a need to strengthen the knowledge bases of smallholder farmers in SSA and adapt their cropping patterns, timing of farm activities and additional crops to the changing conditions within sustainable farming systems.

## 5. The push–pull technology

Cereal stemborers are polyphagous and their host plant range includes other members of the family Poaceae as well as the Cyperaceae and Typhaceae [32–34]. The wild host plants are important not only in maintaining stemborer populations when the cultivated crops are out of season but also for conservation of the pests’ natural enemies. The wild hosts often harbour food sources for many insect pest species and may encourage insect invasion and outbreaks in neighbouring agro-ecosystems [35]. Other reports also indicate the importance of these wild hosts as a buffer against attack of the cultivated crops by some stemborer species [36], implying their role as natural trap plants. Based on previous reports, albeit scanty, on wild habitats as hosts of cereal stemborers in Africa [32,37], scientists at the International Centre of Insect Physiology and Ecology (*icipe*) and their partners, including Rothamsted Research in the United Kingdom, sought to study these interactions from an applied perspective leading to development of an integrated management strategy for these pests. These studies identified the most attractive plant species as trap plants and repellent plants as intercrops. Once these were identified, smallholder farmers selected those that they thought had additional value beyond pest control. Napier grass, *Pennisetum purpureum* Schumach, was selected as the putative trap crop (pull) as it attracted considerably more oviposition by stemborer moths than maize [38,39]. However, emerging young larvae of the stemborers did not appreciably survive on the grass, with over 80% mortality occurring within the first 15 days of larval feeding [39,40]. This is because the grass produces a gummy substance that immobilizes the young larvae as they try to bore into the stem in addition to its low nutritive value for the larvae [40].

Molasses grass, *Melinis minutiflora* P. Beauv, an indigenous poaceous plant with forage value, neither attracted oviposition by stemborer moths nor supported survival of the young larvae. It was therefore selected as a repellent (push) crop and resulted in over 80% reduction in stemborer infestation of maize [38]. Because farmers in SSA often intercrop cereals with legumes, intercropping with forage legumes was investigated and plants in the genus *Desmodium* were found to repel ovipositing stemborer moths [38]. However, it was discovered serendipitously that fields that were intercropped with the silverleaf desmodium, *Desmodium uncinatum* Jacq., had significantly reduced emergence of *S. hermonthica*. This effect was shown in subsequent studies to be significantly superior to that achievable with the food legumes [41]. Effectiveness of the combination of ‘push’ and ‘pull’ components was then tested under on-station and on-farm field conditions and found highly effective in controlling both stemborers and striga resulting in significant yield increases [42–44]. This thus represented the first on-farm report of a combined control of both pest problems using a single method, with concomitant increases in grain yields.

## 6. How the push–pull approach works

The mechanisms by which the ‘push’ and ‘pull’ plants effect such efficient control of the two biotic constraints have been intensely studied and reported. This is important not only to improve our understanding of the mechanisms but also to allow quality control and maintain sustainability in the event that new planting material does not perform as well as the plants originally identified. Furthermore, understanding the mechanisms could allow exploitation beyond the smallholder farming systems for which the technology was originally developed. Semiochemicals released by the companion plants were found to be the key factors mediating the interactions between the plants and the pests. Volatile organic compounds released by the trap plants and maize contain hexanal, (*E*)-2-hexenal, (Z)-3-hexen-1-ol, (Z)-3-hexen-1-yl acetate. Each of these compounds was shown to have positive activity in behavioural tests that investigated oviposition onto an artificial substrate treated with the individual compounds [38]. Subsequent studies showed that trap plants emit significantly higher amounts of the attractive compounds than maize and sorghum [45], and which increase100-fold within the first hour of nightfall, known as the scotophase [46]. This is the time when moths are most actively seeking host plants for oviposition [47]. Although a similar response was observed with maize and sorghum, the increase was approximately 10 times less than in the much more powerfully attractive trap crops.

The repellent intercrops (molasses grass and desmodium), on the other hand, were found to emit volatile organic compounds that were repugnant to the stemborer moths but were attractive to the parasitic wasps and significantly improved their foraging activities [38,48,49]. In behavioural tests, female *Cotesia sesamiae* Cameron (Hymenoptera: Braconidae) were found to be significantly attracted to volatiles emitted by molasses grass [48]. This effect was further confirmed in field trials where plots of maize intercropped with molasses grass recorded significantly higher parasitism of stemborer larvae by *C. sesamiae* [33]. When headspace volatiles from molasses grass and desmodium were analysed, it was found that they contained active compounds that were not in the trap plants. These included (*E*)-4,8-dimethyl-1,3,7-nonatriene, (*E*)-ocimene, β-caryophyllene, humulene and α-terpinolene [38,48,50]. These are semiochemicals produced by plants in responses to herbivore attack and can be important in both direct and indirect effects in plant defense, repelling further pest colonization and attracting the pests’ natural enemies, respectively [51]. Desmodium flowers are particularly highly attractive to *C. sesamiae* [49]. This demonstrates the value of employing intact plants with the inherent ability for constitutive emission of such stimuli in the development of effective crop protection approaches.

**Figure 1.**
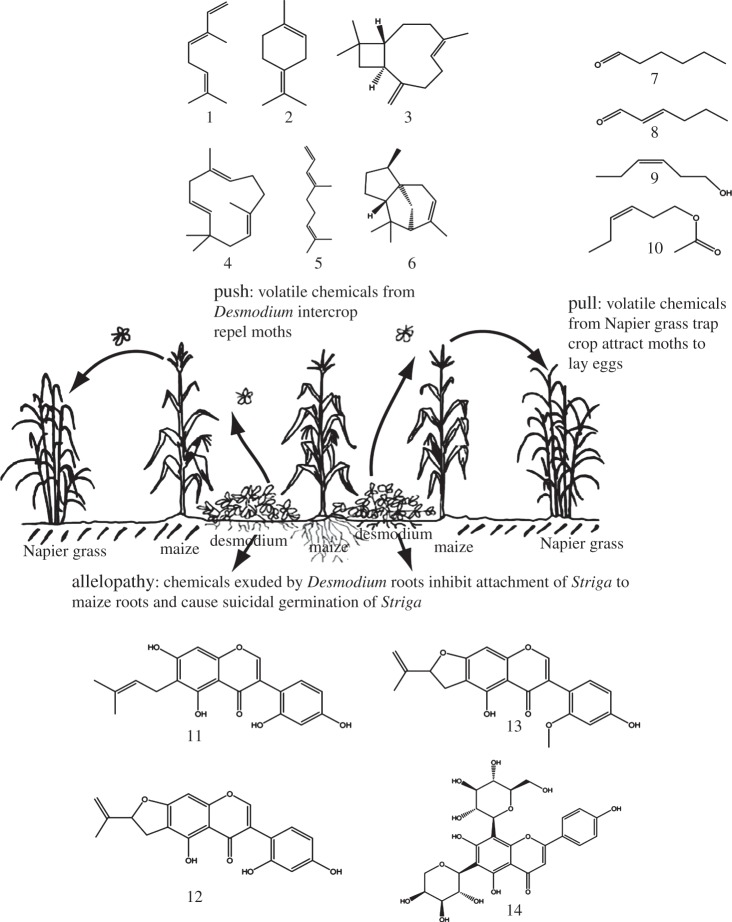
How the push–pull system works: stemborer moths are repelled by intercrop volatiles while attracted to trap crop volatiles. Root exudates from the desmodium intercrop cause suicidal germination of striga and inhibits attachment to maize roots. 1, (*E*)-β-ocimene; 2, α-terpinolene; 3, β-caryophyllene; 4, humulene; 5, (*E*)-4,8-dimethyl-1,3,7-nonatriene; 6, α-cedrene; 7, hexanal; 8, (*E*)-2-hexenal; 9, (Z)-3-hexen-1-ol; 10, (Z)-3-hexen-1-yl acetate; 11, 5,7,2′,4′-tetrahydroxy-6-(3-methylbut-2-enyl)isoflavanone (uncinanone A); 12, 4′,5″-dihydro-5,2′,4′-trihydroxy-5″-isopropenylfurano-(2″,3″;7,6)-isoflavanone (uncinanone B); 13, 4″,5″-dihydro-2’-methoxy-5,4′-dihydroxy-5″-isopropenylfurano-(2″,3″;7,6)-isoflavanone (uncinanone C) and 14, di-*C*-glycosylflavone 6-*C*-α-l-arabinopyranosyl-8-*C*-β-d-glucopyranosylapigenin. Adapted with permission from Khan *et al*. [59]

The striga control effect is mediated by the desmodium intercrop. The mechanisms by which desmodium suppresses striga were elucidated by studying the effects of *D. uncinatum* on *S. hermonthica* in the presence of maize. The effects of *S. hermonthica* are more serious in soils that are degraded and poor in nutrients [17]. Therefore, one of the mechanisms by which *D. uncinatum* suppresses *S. hermonthica* was found to involve the legume's effects on improving soil health, being an efficient nitrogen-fixing legume [52], as well as improving the soil organic matter content [53]. Additionally, because it is a live mulch, *Desmodium* spp. smother weeds including *S. hermonthica*. However, the most dramatic effect involves allelopathic root exudates released by the roots of *D. uncinatum* [54]. These root exudates contain biologically active isoflavanones that stimulate germination of *S. hermonthica* seeds while others and an unusual group of *C*-glycosylflavones inhibit radicle growth [24,25,55,56]. The combination of these compounds provides an efficient way of causing suicidal germination of *S. hermonthica* seeds resulting in depletion of the seed bank in the soil even in the presence of graminaceous host plants [25]. Other species of *Desmodium* spp., including *Desmodium intortum* (Mill.), have also been found to have similar effects on *S. hermonthica* [43,57] and *S. asiatica* [58]. Figure 1 provides a summary of how push–pull works, adapted from Khan *et al*. [59]. With desmodium intercropping, there is also a general reduction in soil temperature and light intensity, resulting in improved soil moisture retention [54], which may in turn prevent striga development, further contributing to reduction of the weed's seed bank in the soil over time [25,60]. Indeed, the density of striga seeds steadily decreases after every cropping season in maize–desmodium intercrops, while in maize monocrop plots it steadily rises [25,61]. Desmodium-based intercrops thus represent one of the very few practical examples of using allelopathy for weed control [55,62].

## 7. On-farm implementation of the push–pull technology

While push–pull as a tool in pest management was first conceived in 1987 [63] and later formalized in 1990 [64], the push–pull technology for control of cereal stemborers described herein is so far the most effective and most widely used by farmers [65,66]. Indeed data from farmers’ fields show effective control of striga and cereal stemborers resulting in significant increases in grain yields. Typically, grain yields have increased from less than 1 t ha^−1^ to at least 3.5 t ha^−1^ for maize [44,67], from less than 1 t ha^−1^ to at least 2.5 t ha^−1^ for sorghum [68] and from less than 0.5 t ha^−1^ to at least 1 t ha^−1^ for finger millet [57]. Moreover, recent data show dramatic effects on striga control [62] with concomitant increase in grain yield of upland NERICA rice through intercropping with desmodium.

Desmodium is an efficient nitrogen-fixing legume [52] and therefore the technology also improves soil fertility through nitrogen fixation, improved organic matter content and prevention of soil erosion [53]. It does not harm soil fauna [50] but improves abundance and diversity of beneficial arthropods [69], partly because there is no usage of insecticides. There is also evidence indicating that higher crop yields and improved livestock production, resulting from the push–pull technology, can support many rural households under existing socio-economic and agro-ecological conditions [70]. This will reduce pressure for human migration into environments needing and designated for protection. Additionally, farmers have mentioned increases in fodder and milk production [71], with an overall improvement in incomes and livelihoods [72,73]. The technology thus opens up significant opportunities for smallholder growth and represents a platform technology around which new income generation and human nutritional components, for example keeping livestock, can be added [74].

## 8. Dissemination and adoption of the push–pull technology

**Figure 2.**
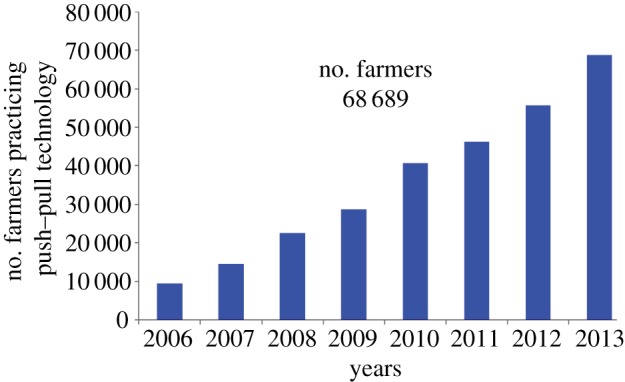
Push–pull technology adoption has significantly increased because of its multiple benefits. (Online version in colour.)

Although push–pull is a knowledge-intensive technology whose effectiveness is dependent on the disciplined establishment and management of the companion plants [40], it is readily adopted and practiced by the smallholder farmers in eastern Africa. To date, the technology has been adopted by over 68 800 smallholder farmers in Kenya, Uganda, Tanzania and Ethiopia (figure 2). About 52 746 adopters are in western Kenya, about 5000 in central Kenya and another 10 600 in Uganda and Tanzania, and 343 in Ethiopia. It is an appropriate system because it uses locally available plants, fits with the tradition of polycropping that smallholder farmers in SSA commonly practice and has multiple benefits. The technology is relevant for most areas in SSA, and is likely to spread further in the region where striga, stemborers and low soil fertility are major constraints to cereal crop production, and where lack of sustainable fodder supply constrains livestock production.

The technology has widely been accepted by farmers as an effective and low-cost technology and its adoption has continuously risen, with an estimated adoption rate of 30% annually. An annual adoption potential of 50% is anticipated because extensive efforts are on-going to transfer the technology to the entire cereal-livestock farming population in SSA. The main drivers of adoption of the technology have been first and foremost to control striga, followed by the need to increase yields of cereal crops, control stemborers, provide fodder, control soil erosion and improve soil fertility [74]. Recently, edible bean production, an important source of plant protein for household nutrition, has been incorporated into the technology thereby expanding its appeal to more smallholder farmers [75]. In addition, push–pull has become a ‘springboard’ for diversifying the smallholder farming system, especially incorporating dairy operations using Napier grass and desmodium as fodder. The farmers have also been able to establish new enterprises such as dairy and poultry farming which are directly benefiting from push–pull products, with poultry benefiting from increased grain yields that serve as feed as well as desmodium leaves that have become an important protein source for these birds. These new enterprises have enabled farmers to start organic farming through preparation and use of animal manure, thus allowing nutrient cycling and reduction in the use of chemical fertilizers [74].

Several factors are responsible for the successful deployment of the technology, with key ones being deployment of a combination of dissemination pathways catering to different socio-cultural and socio-economic contexts of farmers; and multi-level partnerships that allowed exploitation of different individual and institutional capacities. For example, the partnership between *icipe* and Rothamsted Research aided the identification and selection of companion plants, and allowed for elucidation of the science underlying the observed effects of these plants on pests, their natural enemies and weeds, particularly in terms of the active phytochemicals involved [76].

Like any other agricultural technology, challenges of non-adoption have been encountered, mainly attributable to the lack of strong national extension support, lack of information and shortage of inputs, particularly desmodium seed. In addition, although farmers are well able to manage the labour requirement by family members, farmers perceive the labour required for initial plot establishment in the first season to be intensive. To manage this, *icipe* and partners have deployed an intensified dissemination strategy to equip farmers with knowledge that reduces the risk aversion associated with lack of information and therefore builds farmers’ confidence in their decision-making processes. We are enhancing smallholder farmers’ capacity for learning and adoption of push–pull technology through different dissemination pathways. Dissemination of the push–pull technology has been made using the mass media, including radio broadcasts, printed material, agricultural shows, field days (FDs), farmer field schools (FFSs), farmer teachers (FTs) and participatory video. We have recently introduced the use of participatory video technology pioneered by Digital Green of India to disseminate targeted information on the push–pull technology to farmers as it combines social and technical innovation to enable learning, adoption and knowledge sharing among smallholder farmers. For maximum adoption to be reached, the most efficient and economic dissemination pathways have been evaluated and identified as FDs which are likely to lead to a 26.8% increase in adoption if used in farmers’ training, followed by use of FFSs whose probability of convincing farmers to adopt is 22.2% and FTs is at 18.1% [77]. Given these findings, the use of FDs to disseminate the technology has been intensified, initially to train farmers and create interest in the technology, while FFS and FTs are sequentially used to reinforce the messages about the technology. At least 80% of the farmers who attend the initial FD trainings have been shown to adopt the technology [78]. In addition, a multiplier effect has been observed in adoption as a result of farmers’ sharing information through their well-established social capital. FTs rank high, second only to FDs, as a source of information because of the personal contact associated with proximity of smallholder farms, and being part of the social network and therefore able to closely relate with fellow farmers [77]. Through personal contact, each individual farmer is able to influence adoption by an average of about 10 other farmers, and each FT influences adoption by 17 additional farmers [79].

Besides intensive dissemination, efforts are being made to improve the supply, accessibility and affordability of the initially required inputs, specifically the desmodium seeds, through collaboration with seed companies and smallholder farmer groups. Shortage of desmodium seed has been limiting adoption and diffusion of the technology, with its low supply and lack of market development leading to perceived high market prices compared with other seed inputs. This bottleneck is being addressed by initiating large-scale production by seed companies while enabling farmer groups to produce seeds themselves as well as propagate desmodium vines vegetatively. The latter innovation was developed by farmers themselves in central Kenya. The development of the seed value chain with direct participation of private sector players is expected to aid in market development and in turn lead to autonomous technology diffusion as seed becomes more available and affordable. In addition, economic studies of desmodium seed production have been initiated to inform the players in the value chain on appropriate seed pricing, production and marketing costs.

A new strategy that is likely to be an important driver of push–pull adoption is the use of Heifer International's principle of *passing on the gift. icipe* is collaborating with Heifer International, an international non-governmental organization (NGO), to integrate cereal cropping with animal husbandry, in which fodder from the push–pull system is key in sustaining dairy operations, while animal manure from zero grazing units provides organic nutrients for farmers’ fields. Heifer International's method involves recipients of dairy livestock passing on offspring of dairy cows or goats to needy neighbours who carry on the ‘passing on’ process. Using this principle, *icipe* has established a *Nan-Yao Su Desmodium Revolving Fund* (www.push-pul.net/nanyao.shtml) under which it is providing initial desmodium seed to recipients of Heifer International's dairy animals who in turn pass on desmodium seed harvested from their own farms alongside the dairy calves.

## 9. Economic analysis

The economic benefits of push–pull technology have been demonstrated in a series of studies. Khan *et al*. [42] evaluated the benefit cost ratio of introducing push–pull technology compared with the maize monocrop and/or use of pesticides. The study established a positive return on investment of over 2.2 with push–pull technology compared with 0.8 with the monocrop, and slightly less than 1.8 for pesticide use. Push–pull technology using local maize and with no fertilizer had the best gross returns while less profits were registered in the use of fertilizer, implying it was economically propitious to invest in push–pull technology. This was attributed to low soil moisture that affected crop growth and therefore the investment on fertilizer was not recovered.

In a more detailed economic analysis using data over 7 cropping years, returns to investment for the basic factors of production under push–pull technology were evaluated and compared with other cropping methods [72]. In this study, establishment of push–pull technology was associated with extra labour and capital costs (extra labour for planting and maintenance of desmodium and Napier grass and more capital costs in purchase desmodium seeds and Napier grass cuttings) thus high total variable costs were reported for the initial establishment. However, in the subsequent years, the cost significantly reduced contingent upon low land preparation costs and less weeding frequencies as the technology effectively established. Apart from the high initial costs, concerns were also raised on push–pull technology limiting intercropping with edible legumes, for example beans, and also that Napier grass occupied part of the crop land. Despite land being perceived to be lost to trap cropping, the resultant benefits from push–pull technology through maize yield increase and the extra income from sale or utilization of Napier grass and desmodium were more than sufficiently high to cover all the initial capital costs and still make a substantial margin, yet the low investments associated with the other compared technologies were generally not justified by the revenue recovered. Khan *et al*. [72] reported a significant maize yield increase from 0.9 to 1.9 t ha^−1^ in the low potential areas and from 3.9 to 6.3 t ha^−1^ in the higher potential areas. Positive total revenues ranging from $351 ha^−1^ in low potential areas to $957 ha^−1^ in the high potential areas and which generally increased in the subsequent years were also reported. The returns to labour which were recovered within the first year of establishment ranged from $0.5 per man day in the low potential areas to $5.2 per man day in the higher potential areas under the push–pull technology, whereas in the maize monocrop, this was negligible or even negative. Furthermore, the net present value from push–pull technology was positive and consistent over the years. The above findings were corroborated through a study that used discounted partial budget and marginal analysis [73] and concluded that push–pull earned the highest revenue compared with other soil fertility management technologies, including green manure rotation.

## 10. Adaptation to climate change

**Figure 3.**
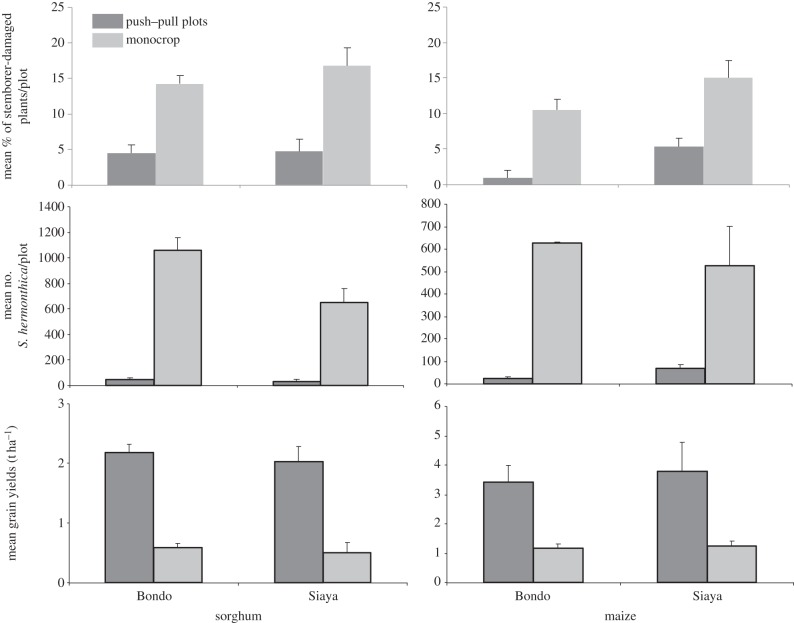
Mean (±s.e.) % stemborer-damaged plants, emerged *S. hermonthica* per 50 maize plants in each plot and grain yields (t ha^−1^) of sorghum and maize planted within a climate-adapted push–pull plot or a sole stand in Bondo and Siaya districts of western Kenya. Means represent data averages of 10 farmers’ fields. In both crops and districts mean % stemborer-damaged plants and emerged *S. hermonthica* were significantly higher in the monocrop than in the push–pull plots. Grain yields were however significantly higher in the push–pull than in the monocrop plots in both crops and districts.

The push–pull technology is effective under a range of different agro-ecologies and with a range of cereal crops, including the more drought-tolerant sorghum and finger millet [57,68]. This makes the technology and its associated benefits relevant currently to 300 million people in SSA, with this number rapidly rising. However, the companion plants are rainfall and temperature limited. Therefore, to extend these benefits to drier areas, and ensure the technology's long-term sustainability in view of the increasingly dry and hot conditions associated with climate change, new drought-tolerant trap and intercrop are being identified. With a recently awarded grant by the European Union, *icipe*, Rothamsted Research (United Kingdom) and African partners in Ethiopia, Kenya and Tanzania, are identifying drought-tolerant companion plants that would deliver similar pest management benefits as the current plants while providing additional economic benefits. Our studies show that *Brachiaria* spp. and particularly the local commercial brachiaria cv mulato can tolerate long droughts of up to three months with no water and more than 30°C (Z. Khan 2013, unpublished data). It is also preferred to maize and sorghum by stemborer moths for oviposition and is preferred by smallholder farmers as animal fodder. Additionally, these studies have demonstrated the beneficial effect of intercropping maize, sorghum and finger millet with the drought-tolerant *D. intortum* on stemborer and striga control, resulting in increased grain yields [68,57]. *Desmodium intortum* withstands drought conditions better and wilts less [80] than *D. uncinatum*. It also has a relatively higher nitrogen-fixing ability, over 300 kg N ha^−1^ yr^−1^ under optimum conditions [52] than *D. uncinatum*, and is therefore more appropriate as an intercrop for the drier areas with more degraded environments vulnerable to further climate change. Therefore, in the adapted push–pull technology, which is currently practiced by about 10 000 farmers in Kenya, Tanzania and Ethiopia, brachiaria cv mulato is used as a trap plant while *D. intortum* is used as an intercrop, with field trials conducted in relatively drier areas of western Kenya with mean annual rainfall of more than 700 mm and mean daily temperatures more than 25°C, indicating effective control of cereal stemborers and striga, with concomitant increases in grain yields in both sorghum and maize (figure 3).

## 11. Pathway to reaching one million households by 2020

Our efforts so far have enabled adoption of the technology by over 55 000 smallholder farmers in East Africa. To reach the target number of one million households by 2020, effective partnerships are crucial. We have established collaborations with the national agricultural research institutes, the national agricultural research and extension systems and other stakeholders, including NGOs. We are expanding and intensifying these collaborations to facilitate dissemination of the technology to smallholder farmers in East Africa and beyond. Sufficient resources will be required, part of which will be mobilized through these partnerships, to improve capacities of national partners in terms of their skill base and material support for wide-scale technology extension efforts.

Concomitantly, we are pursuing the following key strategies aimed at up-scaling the push–pull technology to reach one million smallholder farm families in SSA [76]:

(1) multi-level collaboration with partners including research centres, national extension networks and NGOs, and farmer groups,
(2) deployment of a combination of dissemination pathways catering to different socio-cultural contexts and literacy levels of farmers and
(3) extension efforts underpinned by a robust scientific base and continuous technical backstopping.

Our studies have shown that farmer-to-farmer methods are more effective in technology transfer among smallholder farmers [77,78], with about 80% of those who attend farmers’ FDs sufficiently understanding and adopting the technology. Additionally, each FT is able to recruit an additional 17 farmers within a cropping season (see Dissemination and adoption of push–pull technology section). Therefore, use of a series of interventions involving farmers’ FDs, FTs and FFSs, supported by pathways such as mass media, participatory video, information bulletins and training by specialized extension staff, and public meetings will be intensified to achieve the target.

In addition, human and technical capacities of stakeholders, including national extension systems will have been built for effective and sustainable technology use, thereby enhancing the link between agricultural research and extension programmes. It will additionally establish backstopping expertise in the region thereby responding to beneficiaries’ needs. Moreover, strong linkages and collaborations among stakeholders will be formed and strengthened to facilitate subsequent technology refinement, deployment and resource mobilization and to influence policies designed to improve food security of smallholder farmers.

These efforts will be supported by establishment of an efficient production and distribution system for the required inputs, particularly desmodium seeds, through collaboration with seed companies and their distribution chains, together with smallholder farmer groups. This will also bring on board the phytosanitary and regulatory agencies in the target countries and allow introduction and spread of the technology within and beyond eastern Africa.

It is expected that the intensified technology dissemination efforts above will create a critical mass of one million smallholder farmers using the push–pull technology by 2020 thus allowing its autonomous diffusion beyond the target areas. Expected benefits following adoption of the technology include:

(a) significant increases in grain yields by at least 3 t ha^−1^, and food sufficiency/security achieved through the control of the major abiotic and biotic constrains;
(b) significant improvements in soil fertility, particularly nitrogen fixation, addition of soil humus and prevention of soil erosion, reversal of land degradation, reclamation of abandoned farm land and enhancement of agro-ecosystem integrity in the target areas;
(c) significant improvements in milk and dairy production through provision of year-round quality fodder and improved knowledge on animal husbandry; and
(d) improved livelihoods resulting in better economic and nutritional wellbeing as well as poverty alleviation in the target areas, with the overall contribution towards attainment of MDGs.

Already, the adoption of the technology is having significant impacts on the livelihoods of communities, who are benefiting from better food security, nutrition and health.

## 12. Conclusion

Smallholders in the SSA region have largely not embraced the Green Revolution package of high yielding varieties (HYVs), fertilizers, pesticides and irrigation used in other parts of the world. The HYVs only give high yield under favourable, high-input conditions and if grown under the conditions typical of smallholder cultivation in SSA would often yield less than the traditional farmer varieties [81]. Constraints to production comprise biotic factors (such as pests and weeds) and abiotic factors (such as unpredictable rainfall, land degradation and low soil fertility) while the farmers invest little or no money in inputs. The push–pull system effectively addresses the constraints to production faced by the farmers and is an appropriate system because it uses locally available companion plants rather than expensive imported inputs. Originally devised to control insect pests, it has multiple benefits in controlling striga weeds, improving soil fertility and providing livestock fodder in a truly integrated system. Currently, it is successfully used by 68 689 smallholder farmers mainly in the region around Lake Victoria. However, many millions of smallholder farmers could benefit from it and plans are in place to roll out the technology on a wider scale. Furthermore, the system is being extended to include drought-tolerant companion plants which will make it more resilient in the face of climate change as rainfall becomes increasingly unpredictable. Although the technology is appropriate to African smallholder farming systems, robust science was needed to understand and select the correct plants which released the correct and right amounts of semiochemicals. Technological solutions, for example the push–pull system, are urgently needed to address the real and increasing dangers of food insecurity in SSA.

## Funding statement

The International Centre of Insect Physiology and Ecology (*icipe*) appreciates the core support from the Governments of Sweden, Germany, Switzerland, Denmark, Norway, Finland, France, Kenya and the UK. The work on push–pull technology was primarily funded by the Gatsby Charitable Foundation, Kilimo Trust and the European Union, with additional support from the Rockefeller Foundation, Biovision, McKnight Foundation, Bill and Melinda Gates Foundation and DFID. Adaptation of the push–pull technology to dry weather conditions and to climate change was principally funded by the European Union. Rothamsted Research receives grant-aided support from the Biotechnology and Biological Sciences Research Council (BBSRC), UK, with additional funding provided under the Biological Interactions in the Root Environment (BIRE) initiative.
